# Complete genome sequence of hepatitis B virus identified from a patient suffering from chronic kidney disease in Bangladesh

**DOI:** 10.1128/mra.01151-23

**Published:** 2024-04-16

**Authors:** Md. Golzar Hossain, Mahfuz Islam, Nurejunnati Jeba, S. M. Nazmul Hasan, Marjana Akter, Moslema Jahan Mou, Shyamal Kumar Paul, Sharmin Akter, Mohammad Kamruzzaman Khan, Sukumar Saha, Muzahed Uddin Ahmed, Chirojit Debnath, Md. Arif Uddin Sumon, Md. Salauddin, Chitta Ranjan Debnath

**Affiliations:** 1 Department of Microbiology and Hygiene, Bangladesh Agricultural University, Mymensingh 2202, Bangladesh; 2 Department of Microbiology, Mymensingh Medical College, Mymensingh, Bangladesh; 3 Department of Physiology, Bangladesh Agricultural University, Mymensingh 2202, Bangladesh; 4 Department of Community Medicine, Mymensingh Medical College, Mymensingh, Bangladesh; 5 Department of Medicine, Bangladesh Agricultural University, Mymensingh 2202, Bangladesh; 6 Department of Hepatology, Mymensingh Medical College, Mymensingh, Bangladesh; 7 Department of Microbiology and Public Health, Faculty of Veterinary, Animal and Biomedical Sciences, Khulna Agricultural University, Khulna, Bangladesh; Katholieke Universiteit Leuven, Leuven, Belgium

**Keywords:** hepatitis B virus, genome analysis, chronic kidney disease

## Abstract

Hepatitis B virus (HBV) infection is reported as a risk factor for chronic kidney disease (CKD). In this study, we sequenced the complete genome of an HBV strain identified in a CKD patient in Bangladesh, followed by genomic characterization and mutational analyses.

## ANNOUNCEMENT

The hepatitis B virus (HBV) is a DNA virus that belongs to the family *Hepadnaviridae* and the genus *Orthohepadnavirus,* which primarily infects the liver and can lead to both acute and chronic hepatitis. The HBV genome consists of a relaxed circular DNA of approximately 3.2 kb, which is partially double-stranded. It contains four major open reading frames (ORFs) that encode various viral proteins, referred to as the S, C, P, and X genes (1). In Bangladesh, HBV has a 4% prevalence, and chronic kidney disease (CKD) affects 22.48% of the population (2). Therefore, we sequenced the complete genome of an HBV strain in a CKD patient, providing genomic characterization and mutational analyses.

One patient suffering from CKD was undergoing regular dialysis at the Department of Nephrology, Mymensingh Medical College Hospital, and was found to be positive for HBV as tested by HBeAg, small and large HBsAg ELISA (3, 4). Viral DNA was extracted from the serum sample using the TIANamp Virus DNA/RNA Kit (Tiangen, China), following the manufacturer’s protocol. Quantitative PCR was employed to determine the viral load (Table 1) (3, 4). Serum sample from this patient exhibited a high HBV titer (1.64 × 10^10^ genome copies/mL serum). Traditional PCR was used to amplify the complete overlapping P, S, C, and X genes, as per our established procedures using gene-specific primers (Table 1) (3, 4). The MonoFas DNA Purification Kit (GL Sciences, Inc., Tokyo, Japan) was utilized to purify the PCR-amplified product following the manufacturer’s protocol (3, 4). Sanger sequencing was performed using the same PCR primer to sequence the complete HBV genome using Applied Biosystems BigDye version 3.1, and the reactions were run on Applied Biosystem’s 3730xl DNA Analyzer (Azenta Japan Corp., Tokyo, Japan) (Table 1). A total of six overlapping genome fragments were sequenced, and the minimum quality value score of the sequenced individual base was 20. The sequenced raw data were analyzed and assembled using CLC Sequence Viewer 8. HBV genotype and mutational analysis were conducted using Geno2pheno:hbv with default parameters.

**TABLE 1:** Primers used in this study

HBV genes	Primer names	Sequences (5′–3′)	Product length (bp)	Position
P	EcoRI-Pol-1	GTGGAATTCGGATGCCCCTATCTTATCAACAC	2,499	2,307–3,182, 1–1,623
	Pol-stop-SalI	CACGTCGACTCACGGTGGTCTCCATGCGAC		
S	EcoRI-HBs preS1	GTGGAATTCGGATGGGAGGTTGGTCTTCCAAAC	1,170	2,848–3,182, 1–835
	HBs-stop-SalI	CACGTCGACTTAAATGTATACCCAAAGAC		
X	EcoRI-HBx-1	GTGGAATTCGGATGGCTGCTAGGGTGTGCTG	465	1,374–1,838
	HBx-stop-SalI	CACGTCGACTTAGGCAGAGGTGAAAAAGTTG		
qPCR	HBs F2	CTTCATCCTGCTGCTATGCCT	222	406–627
	HBsR2	AAAGCCCAGGATGATGGGAT		

**Fig 1:**
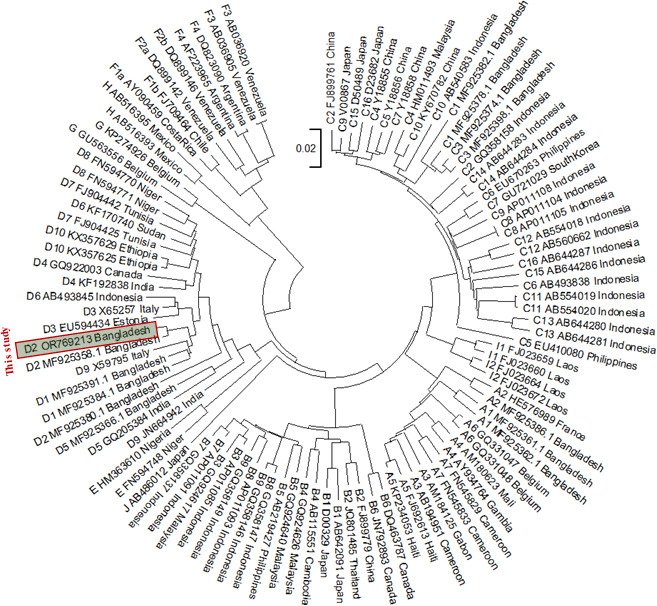
Phylogenetic and evolutionary relationship analysis of identified HBV strain, MGH_HBV-12 (OR769213). Various HBV strain full-genome sequences were obtained from GenBank, aligned, and utilized to construct a phylogenetic tree using MEGA11 employing the neighbor-joining algorithm with default parameters.

The complete circular genome of HBV identified in the patient was 3,182 base pairs in length. The genome is closely related (99.34% and 99.25%) with the HBV genomes identified in Bangladesh (MF925364.1 and LC519789.1) using BLAST. This genome encoded all the expected HBV ORFs, P, S, C, and X with gene lengths of 2,499, 1,170, 639, and 465 bp, respectively. A single A2189T mutation, associated with hepatocellular carcinoma, was found in the PC/core region (2, 5). Two important amino acid changes, A128V and 144E, were found, which are associated with vaccine and/or diagnostic escape phenomena (6, 7). The isolate was identified as genotype D and evolutionarily related to HBV strains from Bangladesh, Italy, and Estonia (Fig. 1). This report underscores the need for further detailed mutational analyses of a larger number of isolates in CKD patients in Bangladesh.

## Data Availability

The complete genome sequence of the identified HBV strain, MGH_HBV-12, has been deposited in GenBank, BioSample, and SRA under the accession numbers OR769213, SAMN39283063, and PRJNA1061811, respectively.
